# Differential Scanning Calorimetry, a Novel Method to Detect Uterine Ischemia-Reperfusion Injury During Autotransplantation in Experimental Sheep Model

**DOI:** 10.3390/biomedicines13102388

**Published:** 2025-09-29

**Authors:** Gabor Fazekas, Balint Farkas, Denes Lorinczy

**Affiliations:** 1 Department of Vascular Surgery, Clinical Center and Medical School, University of Pecs, H-7624 Pecs, Hungary; fazekas.gabor@pte.hu; 2 National Laboratory of Human Reproduction, University of Pecs, H-7624 Pecs, Hungary; 3 Department of Obstetrics and Gynecology, Clinical Center and Medical School, University of Pecs, H-7624 Pecs, Hungary; 4 Department of Biophysics, Medical School, University of Pecs, H-7624 Pecs, Hungary; denes.lorinczy@aok.pte.hu

**Keywords:** uterus transplantation, sheep model, ischemia-reperfusion injury, calorimetry, histology

## Abstract

**Background/Objectives**: A novel treatment of absolute uterine factor infertility is uterus transplantation. In preparation for human surgery, autotransplantation was performed in a sheep model to assess ischemia-reperfusion injury of the uterine wall. **Methods**: Seven multiparous ewes underwent live-donor uterus autotransplantation; in six, the procedure was completed successfully. Tissue blocks of complete uterine wall, endometrium, and myometrium were obtained at four predefined time points: native (baseline), after 1 h of cold ischemia, after 30 min of warm ischemia, and after 30 min of reperfusion. Samples were analyzed by differential scanning calorimetry and routine hematoxylin–eosin histology. **Results**: Histology demonstrated preserved epithelial, glandular, and stromal structures, with only minimal, reversible changes that increased with the ischemic duration. Differential scanning calorimetry confirmed alterations in thermal stability: in the uterine wall and myometrium, the calorimetric enthalpy decreased from baseline (3.40 ± 0.53 J/g) to reperfusion (2.62 ± 0.22 J/g), indicating structural loosening; in contrast, the endometrium calorimetric enthalpy slightly increased, suggesting greater flexibility and less susceptibility to ischemia-reperfusion injury. **Conclusions**: In this preliminary study, differential scanning calorimetry proved to be an effective and sensitive method for detecting early structural alterations in the uterine wall that could negatively impact post-transplant function. Cold and warm ischemia did not cause irreversible damage within a two-hour time frame, supporting the feasibility of short-term preservation in uterus transplantation. The myometrium demonstrated more significant vulnerability than the endometrium, which highlights the necessity of protective strategies to preserve smooth muscle integrity during transplantation.

## 1. Introduction

Absolute Uterine Factor Infertility (AUFI) is a rare condition that affects an estimated 150,000 women in Europe [1]. In these cases, infertility arises from the uterus being either non-functional or anatomically unsuitable for pregnancy. The only options for these patients to have a child are adoption or surrogacy. While adoption is a slow and bureaucratic process with a limited chance to become foster parents, surrogacy is illegal in many countries, for example in Hungary [2]. Uterine transplantation offers these women the only opportunity to give birth to their biological child.

The first live-donor (LD) human uterus transplantation (Utx) was performed in Saudi Arabia in 2000, without extensive prior scientific preparation and experiments. Although the surgery was technically successful, the transplanted organ had to be removed 3 months later due to rejection [3]. Nevertheless, this case has motivated numerous workgroups worldwide, leading to the first successful LD Utx by Brännström and his colleagues in February 2013, which resulted in the birth of the first child born after Utx in September 2014 [4,5].

The key to a successful implementation of the human Utx method is proper preparation; therefore, as reported by Farkas et al. [6,7], our study group has carried out human cadaver and animal surgeries as preparatory steps, while the present investigation reports exclusively on a sheep autotransplantation model. Among many species, the most recommended for preparing human uterus transplantation is sheep, due to the size of sheep’s uterus and the caliber of its supplying vessels being most similar to those of humans [8,9].

Sheep autotransplantation surgeries allow the proper identification of anatomical structures, acquiring perioperative anesthesia, performance of LD uterus autotransplantation, practice of skills for back-table work, and short-term implementation of reperfusion.

A study deals with ischemia-reperfusion injury (IRI) of the uterus during transplantation, confirming a significant impact of IRI on the outcome, including the graft viability, immune response, and pregnancy potential [9]. In cooperation with the Department of Biophysics, the clinics of the Medical School of the University of Pecs have been performing successful thermoanalytical tests on various biological/medical samples for years: human [10] and rat uterus probs [11,12] and rat intestinal tissue in cold/warm ischemia and reperfusion [13,14,15,16]. A comprehensive overview of thermal analysis in biological and medical applications, including DSC principles, representative use cases, and proposed avenues for applying thermoanalytical methods in medical research, is given by Lorinczy (2017) [17].

Differential Scanning Calorimetry (DSC) is a technique used to assess thermodynamic and structural changes in biological tissues by measuring heat flow associated with temperature-dependent phase transitions. In biomedical research, DSC has been successfully applied to detect early molecular and microstructural alterations in tissues subjected to various pathological processes, such as ischemia, inflammation, or degeneration. The broader biomedical validity of DSC as a tissue-level thermoanalytical tool has been demonstrated across diverse applications, including erythrocyte membrane studies, esophageal stent-related changes, cadaveric tendon analysis, and adipose tissue pathology, which supports its relevance in detecting ischemia-related tissue alterations [18,19,20,21]. In the context of uterus transplantation, DSC may serve as a sensitive tool to monitor subtle changes in the integrity and composition of the uterine wall caused by IRI, even before such changes become visible histologically.

Despite previous studies demonstrating that cold ischemia up to 24 h may not cause significant structural damage to the uterus, the exact safe duration remains unclear, and the potential effects of subsequent reperfusion injury are largely unexplored. This study aims to address this gap by assessing ischemia-reperfusion-induced alterations in uterine tissue using DSC and routine histological methods. The hypothesis was that DSC can serve as an effective and sensitive method to detect early microstructural changes in the uterine wall caused by IRI during uterus autotransplantation, and that these changes may influence the success of transplantation. Accordingly, the goal of this study, based on our previous studies [13,14,15,16], is a more precise understanding of the effects of cold and warm ischemia, as well as IRI, on the uterine tissue in a sheep model, which we aim to obtain through DSC and routine histological examinations, and to determine the structural alteration of the entire uterine wall and its layers during the transplantation procedure, which may negatively affect the outcome of the surgery.

## 2. Materials and Methods

This study was conducted at the University of Pecs Preclinical Center. We obtained ethical approval from the Animal Experimentation Scientific Ethics Council (BA02/2000-74/2023). The animals involved in the experiments were treated in accordance with the European Council’s Directive on the protection of animals used for scientific purposes, and this study holds Animal Ethics Committee permission (FB-SEMÁB-B/121/2023; FG-SEMÁB-B/122/2023, KS: KSZ-PTE MÁB/17/B/2023). The surgeries were performed according to the principles of surgical antisepsis and asepsis in a specially designed animal operating room.

### 2.1. Surgical Preparation, Anesthesia

For the surgeries, we used seven multiparous, Landsschaf Merino female sheep acquired from a registered breeder. The animals were declared free of clinically evident diseases; their body mass ranged from 36 to 70 kg, with a mean age of 3.6 years (range: 2–5 years). After transportation, the sheep were housed in a specially designed, stimulus-enriched stall (floor area between 9.29 and 9.66 m^2^) for 48 h to achieve acclimatization, which was followed by 24 h food and 12 h fluid deprivation before surgery. The estrous cycle was neither controlled nor synchronized. The anesthetization followed the protocol described in our prior study [6]. For premedication, the animals were given diazepam (Seduxen, Richter Gedeon Nyrt., Budapest, Hungary) intramuscularly (0.2–0.3 mg/kg), then, while dazed, an 18G peripheral venous cannula was placed on their forelimb, through which they also received diazepam intravenously (0.2–0.3 mg/kg). General anesthesia has been achieved by the administration of thiopental (Thiopental Sandoz, Sandoz Gmbh, Kundl, Austria) (10–20 mg/kg) followed by endotracheal intubation. Narcosis was maintained with sevoflurane (Sevorane, Abbvie PM, North Chicago, IL, USA) (3.0–3.3 etSev%). A central venous catheter was placed in the left jugular vein to ensure an effective and accessible venous route. During the operation, we monitored the animals’ oxygen saturation, blood pressure, and end-tidal carbon dioxide. The animals were protected from hypothermia with a warming blanket and received warmed polyionic isotonic crystalloid infusion (NaCl 0,9% Fresenius, Fresenius SE & Co. KGaA, Bad Homburg, Germany) at 10 mL/kg/h for fluid replacement. At the end of the surgeries, the experimental animals were euthanized with a 40% Euthanimal solution (Alfasan Nederland B.V., Utrecht, The Netherlands).

### 2.2. Surgical Procedure

**Figure 1:**
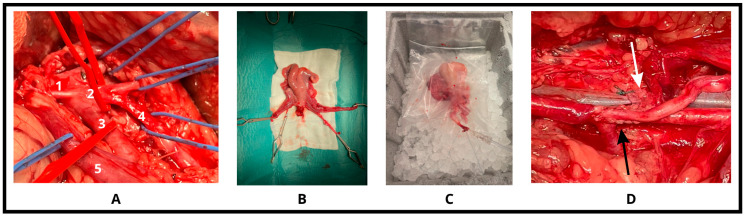
Surgical steps. (**A**) After accurate preparation of the aortic trifurcation and the venous system (1: abdominal aorta, 2: trifurcation, 3: external iliac artery, 4: internal iliac artery, 5: utero-ovarian vein), (**B**) the feeding vessels (2-2 arteries and veins) were ligated, and the organ was removed with bulldog-clamps. (**C**) Following one-hour cold-perfusion on ice, (**D**) the arterial then the venous anastomoses (black and white arrows indicate) were done on both sides to restore the circulation of the uterus.

The surgical plan was based on the method described by Dahm-Kähler et al. [22]. The pelvic anatomy of sheep, particularly the vascular anatomy, shows moderate differences compared to that of humans. The main abdominal artery divides into three branches (trifurcation), then the main artery supplying the bicornuate uterus (uterus bicornis), the uterine artery, originates from the internal iliac artery on both sides, and the main draining vein, the utero-ovarian vein, drains into the internal iliac veins. After dorso-lateral positioning, antiseptic washing, and isolation, the abdominal cavity was opened through a total-median laparotomy, sparing the subcutaneous mammary veins. Once the operator was inside the abdominal cavity, the four-compartment stomach was deflated and intestines were packed into a sterile bag and placed in an extracorporeal position, then the pelvis was exposed using self-retaining and abdominal retractors. On both sides, the broad ligaments were dissected, then the operator proceeded, in the retroperitoneal plane, to dissect the utero-ovarian vein up to its origin on both sides. The ureters were identified and elevated with a rubber loop. The origin of uterine arteries was prepared and dissected in antegrade direction, respecting the ureters. Before organ removal and clamping of the vessels, 10,000 IU of Na-Heparin (Heparinbene Na 25,000 NE, Teva Zrt. Debrecen, Hungary) was administered intravenously to prevent clot formation. The vessels were ligated distally from the organ, clamped proximally, and resected with 6–8 cm long remaining vascular pedicles. After exposing the recto-uterine and vesico-uterine spaces, hysterectomy was performed. The removed organ was placed on a sterile table for back-table work. One horn of the uterus was resected, then the contralateral uterine artery was cannulated and flushed with constant pressure (5000 IU Na-Heparin/500 mL 0.9% NaCl solution) until clear fluid appeared on the venous side. After completely washing out the blood, the organ was subjected to cold ischemia, maintaining continuous perfusion with the same solution, and placed in a sterile organ bag at 4 °C ice water for 60 min. An hour later, the perfusion was stopped and the organ was autotransplanted. We first performed cervico-vaginal anastomosis with an absorbable continuous suture for organ orientation. During uterus perfusion, the external iliac artery and vein were dissected on the ipsilateral side, to prepare for implantation. After orientation, first the venous, then the arterial “end-to-side” anastomoses, were performed with 6/0 Optilene (B. Braun, Melsungen, Germany) continuous suture. Following successful restoration of circulation, the transplanted uterus regained its color and temperature, and we observed bleeding due to incision of the wall, which indicated satisfactory circulation (see Figure 1).

### 2.3. Sampling

In all 6 successful procedures, three different types of tissue blocks (complete uterine wall, endo- and myometrium) were removed at the same surgical timepoint. The first samples were removed freshly from the native uterus before vascular clamping. The second samples were cut out following 1 h of cold ischemia and the third specimens were obtained after an additional 30 min of warm ischemia. The fourth tissue blocks were excised for 30 min following the total restoration of uterine circulation. These samples were analyzed by DSC, and light microscopy examinations were performed using hematoxylin–eosin (HE) stains.

### 2.4. Light Microscopy Evaluation

Samples were fixed for 24 h in 10% neutral buffered formalin, in support of routine histological examination. Tissue blocks were embedded in paraffin, and 2–4-micrometer-thick sections were sliced and stained by applying HE dye. HE-stained histological sections of the uterus, after warm and cold ischemia as well as following implantation, were compared to native samples, analyzed using Case Viewer software (3DHistec Ltd., Budapest, Hungary), and evaluated by two independent pathologists.

### 2.5. DSC Measurements

The thermal denaturation of the samples was investigated with a SETARAM Micro DSC-III (Caluire-et-Cuire, France) calorimeter. In the calorimeter, a heatable/coolable metal block contains two containers (sample and reference vessels), which can be heated/cooled at a rate of 0.001–1.2 K min^−1^. The reference usually contains the buffer of the biological/medical sample, or a material with comparable heat capacity and mass. A control system maintains zero temperature difference between the two containers and the heating program during the process. The heat transferred during heating increases the internal energy (and therefore the temperature) of the system. As soon as the disordered thermal motion (vibration amplitudes/rotation speed) in the biological sample becomes so large that the connection between the individual macromolecular units can be broken, from there on, the internal energy does not increase, but the transferred heat causes a structural change, so that the temperature of the tested sample lags behind the reference. From this moment, the control system feeds extra energy into the sample container to sustain zero temperature difference in the case of an endothermic process (and into the reference in the case of an exothermic process). This energy (heat flow) is equal to the energy required for the structural change, which is registered as an output signal in the function of temperature/time. Each analysis was performed within the range of 0–100 °C with a heating rate of 0.3 K min^−1^. Conventional Hastelloy batch vessels (V_max_ = 1 mL) were applied for the experiment, with an average sample mass of ~150 mg (range: 110–200 mg). Normal saline solution was used as a reference. The reference and sample vessels were equilibrated with a precision of ±0.1 mg, so heat capacity correction between the sample and reference was not needed. The second scan of the denatured samples was used to make the baseline correction. The melting/unfolding temperatures of the samples (T_m_) were defined as the peak of the DSC scans (at this point 50% of samples turn from native into denatured/unfolded state). To characterize the interaction between the components of a macromolecular system, we use the temperature range (T_1/2_) corresponding to half of the maximum heat flow (measured at T_m_). A low value of this metric indicates a strong interaction, while a high value indicates a loose interaction between the thermal domains of the system. The calorimetric enthalpy change (ΔH_cal_) was calculated from the area under the heat absorption curve with the SETARAM two points setting software. The wet sample mass in grams was used to normalize the ΔH_cal_ values (in Jg^−1^ unit). The change in heat capacity (ΔC_P_) was plotted as the heat flow difference (ΔQ) between the native and denatured state (its value can be determined as ΔH divided by the heating rate). DSC measurements were performed by an unblinded operator using predefined intraoperative sampling time points; randomization was not applicable.

## 3. Results

In six out of seven cases, the operations were successful. In our first attempt, the sheep succumbed during the surgery due to unexpected anesthetic complications (respiratory arrest). We improved our technique and, thus, from the second procedure onwards, the removal, “back table” preparation, autotransplantation, and sample collection were uneventful.

### 3.1. Light Microscopy Evaluation of Uterus Wall

**Figure 2:**
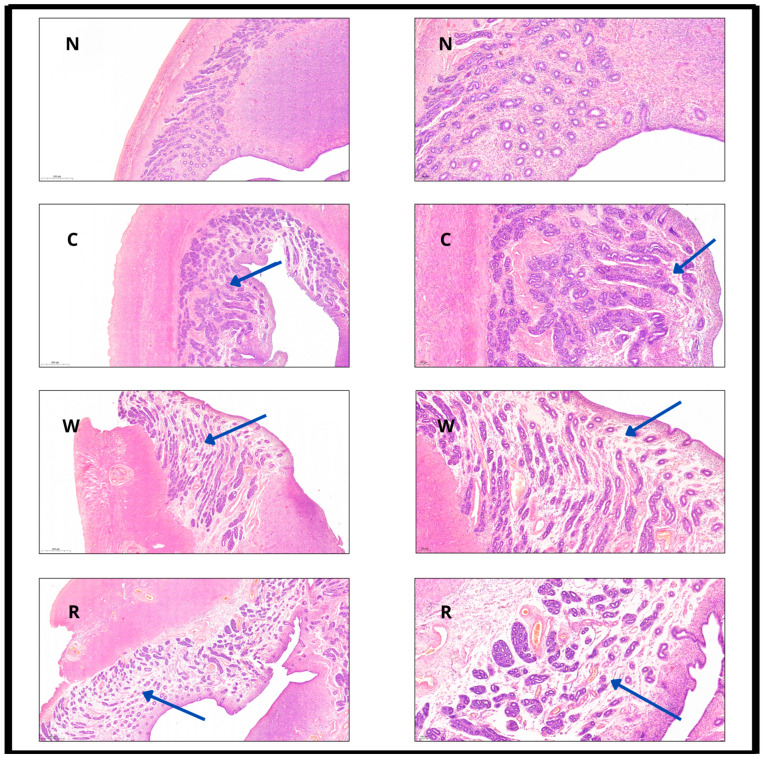
Hematoxylin–eosin (HE) staining of the uterine wall at different timepoints with 4× (left column) and 10× (right column) magnification, scale bars included. N: normal; C: after cold ischemia; W: after warm ischemia; R: after reperfusion. Images in the left column demonstrate mild transmural structural change, while images in the right column present the preservation of columnar epithelium and the edematous “loosening” of the stroma (blue arrows indicate stromal edema and structural loosening across the different time points).

Hematoxylin–eosin staining was performed for routine histological examination to compare each sample (cold, warm ischemia, and reperfusion) to freshly excised, native uterine wall. The two functional layers of uterine wall (endo- and myometrium) showed no significant changes in their tissue structure or cellular level throughout the samples. The normal native uterus is lined by the endometrial mucosa, consisting of a simple columnar epithelium, which forms tubular glands within a stroma rich in small vessels. This epithelial layer and the glands were preserved and remained regular. The nuclear staining of the epithelial layer did not indicate (no fading) damage after the ischemia and/or reperfusion, and the same was observed in the other layers as well. There was also no identifiable sign of damage in the surrounding layer, as confirmed by the absence of reduced cell staining (cellular nucleus and cytoplasm) and the retained tissue structure. Only a minimally decreased regularity and staining intensity were demonstrated following reperfusion in the smooth muscle; however, this is quite normal and reversible after ischemia. The most characteristic alterations were the “loosening” and edema of the structure of the stroma and the dilation of the stromal small vessels (containing red blood cells) as a consequence of Ischemia-reperfusion injury. These changes became more pronounced as the ischemic time increased (cold ischemia -> warm ischemia -> reperfusion) and were most prominent in the reperfusion sample (see Figure 2).

### 3.2. DSC Evaluation

**Figure 3:**
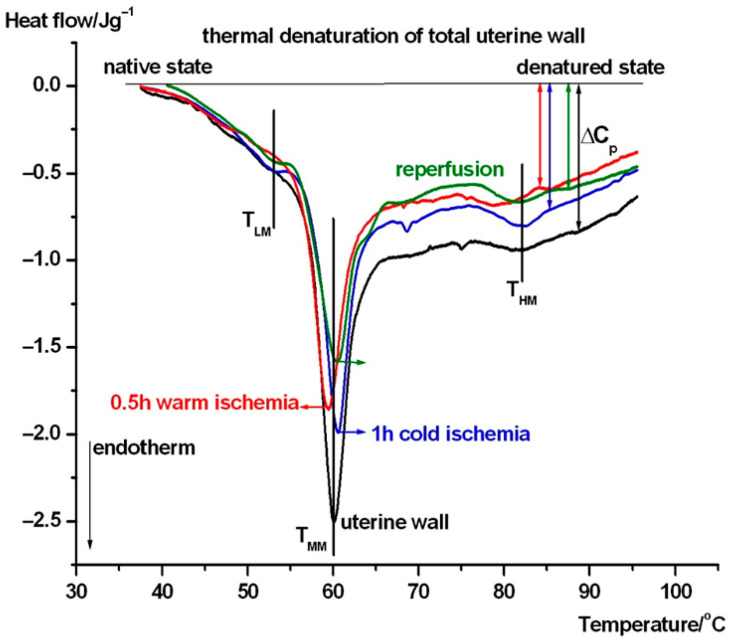
Thermal denaturation of complete uterine wall and after its treatment. The DSC scans are averages of six experiments (T_LM_, T_MM_, and T_HM_ stand for the melting or denaturation/unfolding temperature in a low, middle, and high temperature range. The heat flow is normalized on the wet mass of samples. Endotherm deflection downwards).

**Figure 4:**
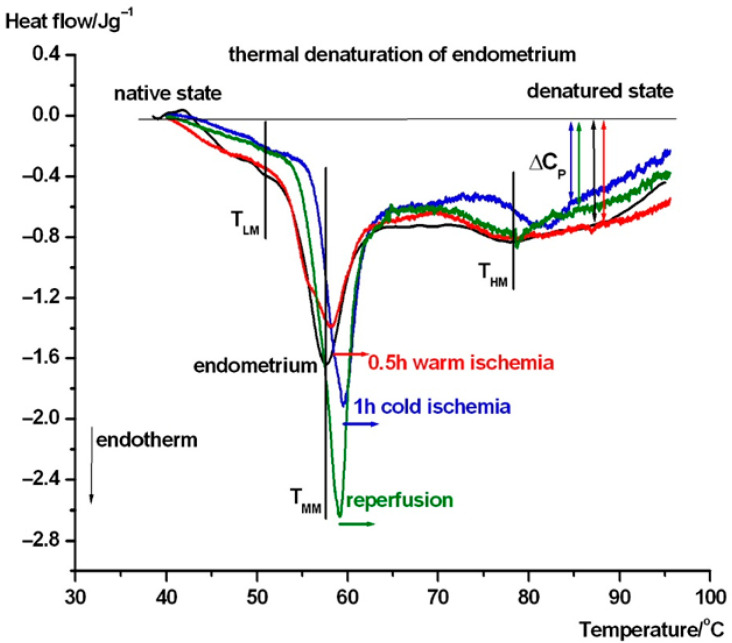
DSC scans of endometrium and after its treatment. The symbols are as in Figure 3.

**Figure 5:**
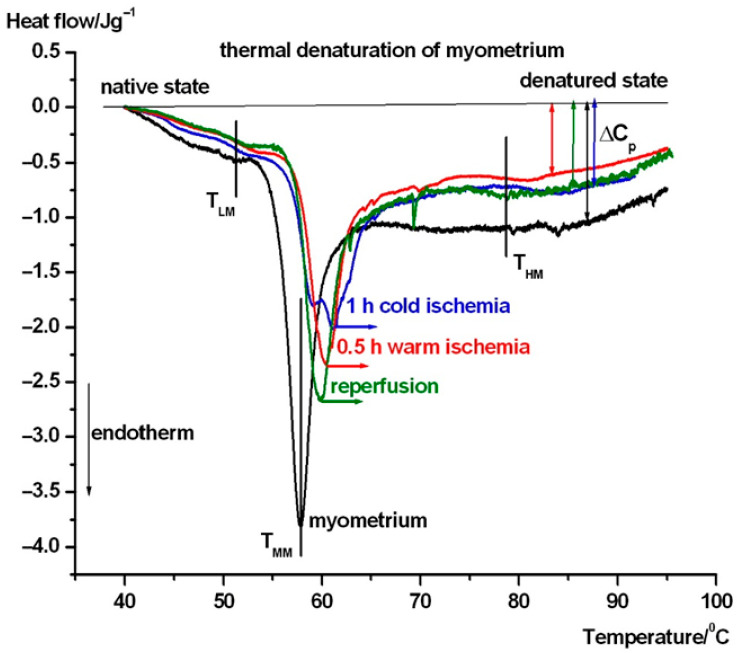
Running of DSC scans during the thermal denaturation of myometrium in different stages of transplantation protocol. The symbols mean the same as in Figure 3 and Figure 4.

In Figure 3, the average thermal denaturation of complete uterine wall normalized to wet mass in the intermediate phases of transplantation steps can be seen. Figure 4 and 5 show the same in the case of endometrium as well as myometrium. We have summarized the most characteristic thermodynamic parameters in Table 1. The difference between the native and transplanted states is immediately apparent from the course and shape of the DSC scans. Below, we summarize the visible differences between the different samples.

In case of the untreated uterus samples (see Figure 3), a weak pretransition can be seen at ~47 °C (T_LM_), and it shifted to 53.8 °C with the contribution of myometrium (see Figure 5 and Table 1) as part of the complete wall, and also shifted after cold ischemia and reperfusion, but it is poorly visible in warm ischemia. The middle range (T_MM_) of ~60 °C exhibits a weakening (decrease by ~1 °C) of the wall in warm ischemia, while a mild strengthening (increase by ~1 °C) was observed after cold ischemia and reperfusion based on the denaturation temperature. At the higher temperature range, the denaturation contribution of endometrium (see Figure 4 and Table 1) can cause a shift and fluctuation in T_HM_ between 79 and 82 °C in case of a complete wall (1 h cold ischemia and reperfusion, while after 0.5 h warm ischemia it remains ~79 °C (see Figure 3)). Double arrows indicate the change in the heat capacity of the samples at the end of the endothermic processes at higher temperatures, referring to the native stage. This parameter shows a decreasing trend across treatments, which suggests that ischemia and reperfusion relaxed the uterine wall.

We have performed the same test in the case of endometrium (see Figure 4). Around 51 °C (T_LM_), mild endotherm appears, which shifted to 52 °C in cold ischemia and disappeared in warm ischemia and reperfusion. The T_MM_ shifted down to ~57.7 °C compared to uterus wall, and after ischemia and reperfusion, it increased by ~1-2.5 °C, exhibiting a stabilizing structural effect of these procedures. The T_HM_ is at ~78 °C except for in cold ischemia, when it is ~82 °C. It seems that cold ischemia causes a shock, which manifests as a more stable structure. The heat capacity of the samples at the end of the endothermic processes at higher temperatures exhibit stronger structural flexibility only after cold ischemia and reperfusion. The scan of 0.5 h warm ischemia runs practically parallel with the native endometrium sample.

The same thermal denaturation tests of myometrium exhibited very mildly elevated endotherm (T_HM_) processes (in the average scans, e.g., in case of cold and warm ischemia, it can be the consequence of imperfect preparation of this part from the uterine wall (see Figure 5)). The T_LMs_ appeared in the same range as in the case of uterine wall and was higher than in endometrium, and there was a definite shift to a higher temperature in case of transplantation steps. The T_MM_ also exhibited a significant increasing shift in reperfusion-warm and -cold ischemia compared to endometrium and intact uterine wall. The heat capacity change was the smallest in warm ischemia, while cold ischemia and the reperfusion caused the same, slightly higher change. The intact myometrium produced the highest one, which means that, after the denaturation, it remains in the more compact structure.

It can be seen from Figure 3, Figure 4 and Figure 5 that the main thermal events (signed by T_MM_) in the case of uterine wall fluctuate around 60 °C, independently of the actual stage of transplantation. Endometrium and myometrium show stronger ischemia and reperfusion dependence.

T_M1/2_ is a sensitive parameter of heat-induced changes: uterine wall becomes a more ordered structure after cold ischemia, while, after reperfusion, it becomes more elastic (more relaxed). The endometrium is more densely packed after reperfusion and much looser after warm ischemia. Myometrium is most ordered in its native state, while the cold ischemia exhibits a large loosening (reduced cooperativity) of its structure.

In comparing the characteristic denaturation temperature parameters, the calorimetric enthalpy shows a clear decrease after the interventions in the entire uterine wall and myometrium, but an increase in the endometrium. This can be interpreted as indicating that the myometrium has a stronger influence on the thermal behavior of uterus wall than the endometrium.

## 4. Discussion

Numerous publications in the literature have focused on animal studies on Utx, which has resulted in an extensive knowledge of the field [23,24,25,26,27,28]. The Utx procedure has been investigated in rodents (rats and mice) [23,24], in domestic animals such as pigs [25], and in nonhuman primates such as baboons [26] and rhesus macaque [27]. Almost all animal models show limitations. For instance, rodent surgeries require microsurgical skills and are hard to perform. In pigs, the vessels that enable anastomosis are small and found deep in the narrow pelvis, which results in low surgical success rates [28]. Nonhuman primates theoretically would be optimal candidates for Utx experimental models, due to the anatomic and physiologic similarity of their reproductive organs to those of humans, but despite the International Federation of Gynecology and Obstetrics (FIGO)’s indication that human UTx should be performed after significant and adequate research in appropriate large animal models, including primates [29,30], ethical concerns are still raised on nonhuman primate studies. Another limitation of such surgeries is the small and narrow pelvis of these animals, which makes the surgeries extremely challenging due to limited access to candidate vessels for anastomosis. On the other hand, the sheep model demonstrates some considerable advantages compared to other models. The animals are large enough to have vessels that enable proper vascular anastomosis, the vessels feeding the uterus have similar diameters to those of humans, the pelvis is easily approachable, and, despite having a bicornuate uterus, the size of the reproductive organs are identical to those of humans. Taking all of this into account, the sheep model is the most widely investigated one, and teams from UK, Israel, France, and Hungary have all used it for preparation for human Utx trials [6,8,31,32].

Success in Utx can be determined in two ways. One way is surgical success, which is measured by the full recovery of the circulation of the transplanted organ in the short term and the absence of rejection in the long term, which is generally determined in relation to the vascular anastomoses, the venous outflow of the transplanted organ, and the efficacy of the immunosuppression therapy. The other way of measuring success, which is, from the recipient’s perspective, the most important aspect of Utx, is the function recovery after surgeries, which means the achievement of fertility. In the sheep model, both non-rejecting autologous and rejecting allogenic Utx experiments were carried out [33,34]. In autologous experiments, immunosuppression therapy is not required, and natural mating is also possible. It results in a 75% conception rate and the delivery of normal-sized offspring via cesarean section [33,35]. This was the main reason why we chose this model for our current experiments.

In humans, the most frequent antepartum obstetrical complications are preterm delivery (31.3%) and preeclampsia (28.1%) [36]. Preeclampsia is a rare complication of pregnancy that exclusively appears in humans. Two major subtypes are known: the early- and late-onset types. The early-onset subtype is clinically more severe, and abnormal placentation that originates from epithelial damage of the microvasculature at the feto–maternal interface is suspected in the background [37]. It is theorized that IRI damage may play a role in the development of the antepartum manifesting condition, which puts Utx-IRI investigation into another spotlight.

In our current preliminary study, we demonstrated for the first time that IRI-induced microstructural changes can be detected using DSC, accompanied by histological examination. Our main goal was to determine whether this novel methodology could be helpful in clinical application and provide more information about the microstructural alterations during the entire transplantation process. Our histological samples, using routine HE stains, demonstrated only minimal changes in the structure of the uterine wall and its two functional layers, with restored epithelial, glandular, and stromal tissues (endometrium), as well as reversible, non-substantial irregularities among smooth muscle fibers (myometrium). The edema formation and structural loosening in the stroma, along with their intensification over ischemic time, as well as the progressive dilation of the feeding vessels, are normal reactions during ischemia. In the absence of cellular damage, these changes are likely reversible and, following the onset of regeneration, they are presumed to recover and will not affect future function. However, ischemic time is an important factor for preserving structural integrity, and did not exceed 2 h throughout our surgeries.

The DSC scans showed that calorimetric enthalpy of uterine wall decreases during ischemia and reperfusion, such as myometrium, and this suggests a loosening of the structure of complete uterine wall and myometrium. In the case of endometrium, enthalpy showed a trend of growing, which suggests greater structural flexibility during the process. Thus, the endometrium shows less dependence on IRI. Ischemia has a more pronounced effect on the structural integrity of myometrium, which proved to be the dominant factor in the IRI of the whole uterine wall.

Layer-specific DSC signatures—particularly the myometrial decrease in ΔH with shifts in Tm/T1/2—support defining tighter preservation windows, minimizing warm ischemia, and optimizing cold storage (solution, temperature, duration). These data also motivate testing smooth-muscle-protective strategies (e.g., tailored additives, controlled reperfusion/machine perfusion). The observed thermal changes are compatible with early protein unfolding and altered cooperativity among cytoskeletal and extracellular-matrix components under IRI, together with possible mitochondrial contributions. This is consistent with the greater myometrial vulnerability relative to the more flexible endometrium and helps explain the layer-specific DSC patterns that were observed. In practice, DSC can serve as a sensitive quality-control readout alongside histology and functional assessment during protocol development for human UTx. Through this investigation, we aimed to simulate the process of LD transplantation in general, applying an approximately similar duration of cold ischemia and a slightly shorter period of warm ischemia. Considering the experiments, our results suggest that IRI caused by cold and warm ischemia within this timeframe (ca. 2 h) does not result in irreversible structural damage in the uterine tissue.

Previous investigations have also attempted to determine the cold and warm ischemic tolerance of the uterus, which is an important factor in organ transplantation. Most of the available data come from rodent models. In one animal study, the tolerability of the mouse uterus was investigated, and it was demonstrated that 24 h of cold ischemia in University of Wisconsin solution does not lead to irreversible morphological changes in uteri; however, 48 h preservation resulted in tissue necrosis after transplantation [38,39]. In another experiment, uteri subjected to 36 h of cold ischemia demonstrated long-term survival and preserved fertility, which proved that the uterus can tolerate 36 h of cold ischemia [40]. These studies confirmed that 48 h of cold ischemia causes irreversible structural and functional damage to the uterus. Warm ischemic time was investigated in rats by Wranning et al., who confirmed that prolonged warm ischemia negatively affects uterine tissue viability [38,41].

In a sheep model, normal uterine contractility and normalized lactate levels were observed after 1 h of reperfusion, following 1 h of cold and 3 h of warm ischemia [22,38]. A French experiment in ewes suggested that the uterus has good tolerance to extended cold storage prior to transplantation. After 24 h of cold ischemic preservation, viable uteri were confirmed both macroscopically and histologically following autotransplantation [42].

To date, the exact and tolerable duration of cold ischemia in the human uterus has not been clearly identified. The only established fact is that the tolerance of human myometrial tissue against cold ischemia is at least 6 h in an appropriate preservation solution [38,43]. Furthermore, prolonged cold ischemia of up to 24 h in Celsior preservation does not lead to significant morphological or structural damage, in contrast to 48 h long preservation [38,44]. However, the potential harmful effects of reperfusion following ischemia have not been examined in these studies [38].

Our study has several limitations, including the low number of animals and the fact that the estrous cycle was neither controlled nor synchronized in the examined sheep. Cycle-related hormonal variation in sheep can influence uterine perfusion and tissue properties, potentially modulating the severity of reperfusion injury and DSC parameters. Regarding the small sample size, this preclinical investigation was a proof-of-concept, preliminary exploratory study relying on descriptive statistics. Moreover, there is usually a gap between the findings in animal and human studies. In addition, the absence of operator blinding for DSC constitutes a potential source of measurement bias. In our study, we made a genuine attempt to assess the microstructural changes of uterine tissue using DSC, following cold and warm ischemia and reperfusion in correlation with histological findings. Whereas previous ovine studies primarily relied on macroscopic reperfusion signs, contractility, lactate normalization, and routine histology [22,42], our work introduces DSC to determine microstructural alterations during IRI. It has been proven to be an effective and sensitive method to detect the early structural alterations that may affect later function. To gain a better understanding and to determine the tolerance time of the uterine wall more precisely against cold and warm ischemia, further investigations with larger sample sizes and extended ischemic storage times are needed.

## 5. Conclusions

In this study, we demonstrated for the first time that IRI-related microstructural changes in the uterine wall can be effectively detected using DSC, supported by histological analysis. Our findings suggest that cold and warm ischemia, within a clinically relevant timeframe of approximately 2 h, do not cause irreversible structural damage to the uterus. DSC proved to be a sensitive tool for detecting early alterations, and the results suggest that the myometrium has a more pronounced impact on the thermal behavior of the uterine wall than the endometrium, indicating its greater susceptibility to ischemia-reperfusion injury. These results support the feasibility of short-term cold and warm ischemic preservation in uterine transplantation and highlight the potential applicability of DSC analysis in clinical practice within this field. However, further studies with larger sample sizes and extended ischemic times are needed to better define the tolerance limits and improve clinical applicability.

## Figures and Tables

**Table 1:** The more characteristic thermodynamic parameters of denaturation of uterine wall and its compounds during the transplantation steps. Symbols: T_LM_ stands for denaturation temperature of lower transition, T_MM_ is middle melting, T_M1/2_ is its temperature interval at the half-maximum heat flow (where ~ 50% of macromolecules are in unfolded state), which informs us about the change in the thermal stability of the system, T_HM_ is the highest denaturation temperature, and ΔH_Mcal_ is the calorimetric enthalpy change during the main denaturation. Data are averages with ± s.d. and rounded to one (temperature) or two decimal places. Bold numbers refer to significant change, blue arrows represent decreasing and red ones increasing tendency of ΔH_cal_.

Sample n = 6	T_LM_/°C	T_MM_/°C	T_M1/2_/°C	T_HM_/°C	ΔH_Mcal_/Jg^−1^	
**Uterine wall**	47.5 ± 0.2	60.2 ± 0.6	3.8 ± 0.2	82.4 ± 0.5	3.40 ± 0.53	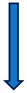
1 h cold ischemia	**53.8 ± 0.4**	60.9 ± 0.5	**3.2 ± 0.2**	82.4 ± 0.5	3.18 ± 0.24	
0.5 h warm ischemia	-	59.9 ± 0.3	3.6 ± 0.3	**78.7 ± 0.4**	2.69 ± 0.31	
0.5 h reperfusion	**53.8 ± 0.4**	60.6 ± 0.3	4.2 ± 0.3	82.4 ± 0.5	**2.62 ± 0.22**	
**Endometrium**	51.1 ± 0.4	57.7 ± 0.8	4.3 ± 0.3	78.6 ± 0.4	2.23 ± 0.10	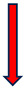
1 h cold ischemia	52.0 ± 0.4	59.8 ± 0.8	**3.6 ± 0.2**	80.0 ± 0.5	**2.75 ± 0.23**	
0.5 h warm ischemia	-	58.4 ± 1.0	**5.7 ± 0.3**	78.6 ± 0.4	**2.74 ± 0.25**	
0.5 h reperfusion	-	59.1 ± 1.0	3.2 ± 0.2	78.8 ± 0.4	**2.68 ± 0.21**	
**Myometrium**	50.8 ± 0.3	56.9 ± 1.2	2.5 ± 0.2	-	3.51 ± 0.98	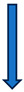
1 h cold ischemia	**52.6 ± 0.4**	60.1 ± 0.2	**5.6 ± 0.3**	84.4 ± 0.5	2.94 ± 0.50	
0.5 h warm ischemia	**53.9 ± 0.5**	60.3 ± 0.5	**3.3 ± 0.2**	**81.2 ± 0.4**	3.20 ± 0.39	
0.5 h reperfusion	**52.6 ± 0.4**	59.8 ± 0.5	**3.3 ± 0.2**	**78.8 ± 0.4**	2.49 ± 0.53	

## Data Availability

Data is unavailable upon request from the correspondent author.

## Abbreviations

The following abbreviations are used in this manuscript:

AUFI	Absolute uterine factor infertility
Utx	Uterus transplantation
LD	Live donor
IRI	Ischemia-reperfusion injury
DSC	Differential scanning calorimetry
HE	Hematoxylin–eosin
FIGO	International Federation of Obstetrics and Gynecology
